# Beyond Diabetes: A Review of Emerging Indications for Glucagon-Like Peptide-1 Receptor Agonists

**DOI:** 10.31083/RCM44528

**Published:** 2026-01-22

**Authors:** Lucianne West, Harsh Patolia, Brittany Chapman, Luke Laffin, Amanda R. Vest, Andrew J. Sauer, Trejeeve Martyn

**Affiliations:** ^1^Department of Transplant Surgery, Tufts Medical Center, Boston, MA 02111, USA; ^2^Department of Cardiovascular Medicine, Heart Vascular Thoracic Institute, Cleveland Clinic, Cleveland, OH 44195, USA; ^3^Kaufman Center for Heart Failure and Recovery, Cleveland Clinic, Cleveland, OH 44195, USA; ^4^Saint Luke’s Mid America Heart Institute, Kansas City, MO 64111, USA

**Keywords:** glucagon-like peptide-1, GLP-1 receptor agonists, obesity management, cardiovascular outcomes, renal protection, metabolic-associated steatotic liver disease, weight loss pharmacotherapy

## Abstract

Glucagon-like peptide-1 receptor agonists (GLP-1 RAs), originally developed for glycemic control in type 2 diabetes, have emerged as transformative agents with broad therapeutic applications across multiple organ systems. This review explores the expanding role of GLP-1 RAs in managing cardiometabolic diseases, including obesity, heart failure (particularly with preserved ejection fraction), chronic kidney disease (CKD), and metabolic dysfunction-associated steatotic liver disease (MASLD). Robust clinical trial data support the efficacy of GLP-1 RAs in promoting weight loss, improving cardiovascular outcomes, and preserving renal function, with additional trials underway to further strengthen and expand the evidence base. Despite the growing utility of GLP-1 RAs, challenges related to cost, access, adherence, and implementation persist, particularly for indications beyond diabetes. However, innovations such as oral formulations and combination therapies may help improve accessibility and sustained use. As clinical guidelines evolve, targeted integration of GLP-1 RAs into care models may transform the prevention and treatment landscape for complex, chronic diseases.

## 1. Introduction

**Fig. 1.**
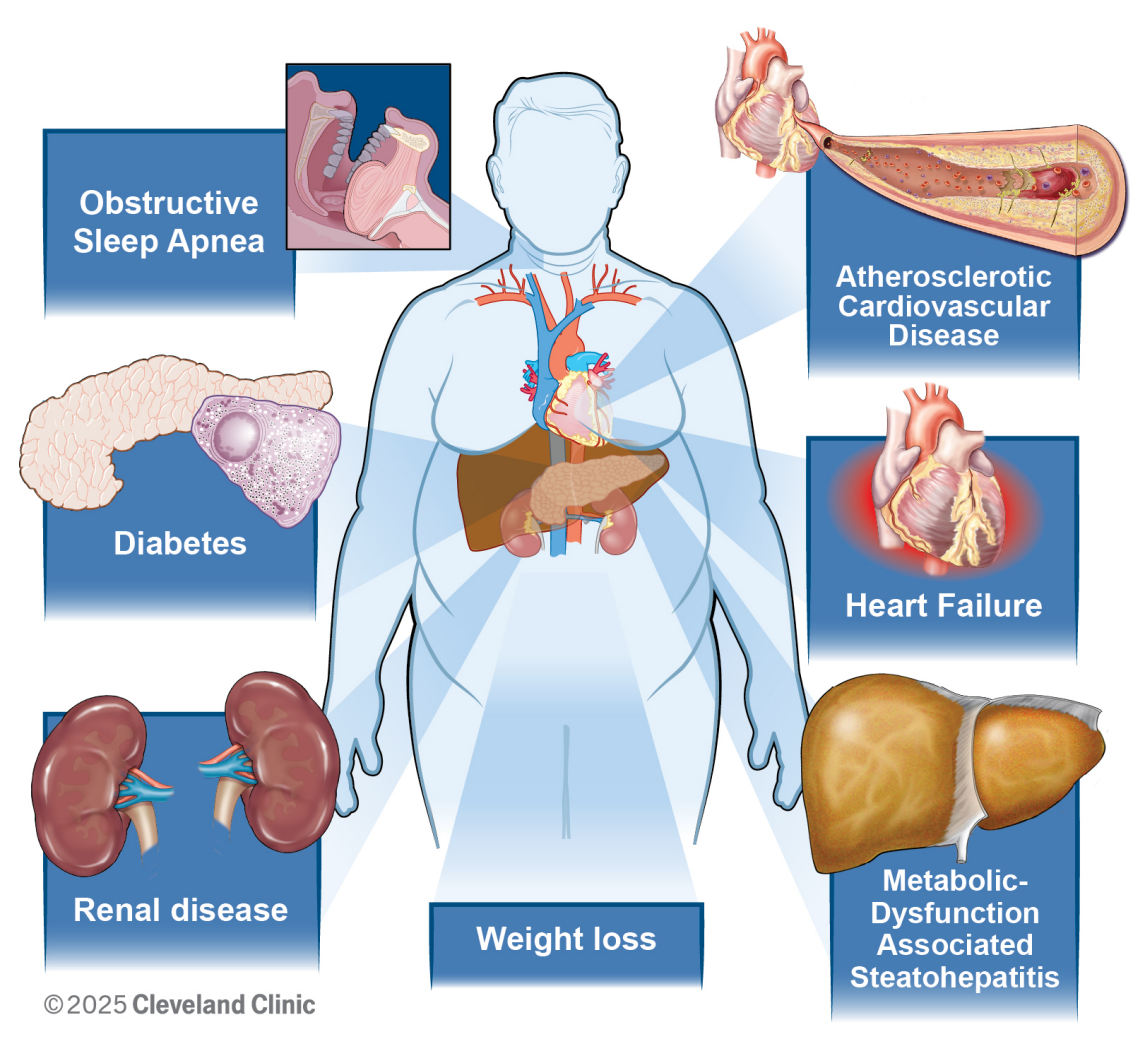
**Emerging indications for glucagon-like peptide-1 receptor agonists**.

Glucagon-like peptide-1 receptor agonists (GLP-1 RAs), among other incretin-based therapies, have gained significant attention over the past 5 years. Initially introduced to improve glycemic control in patients with type 2 diabetes, the benefits of GLP-1 RAs now extend well beyond glucose regulation. GLP-1 RAs exert a variety of beneficial effects with mechanisms of action that influence cardiovascular, renal, hepatic, and metabolic systems. These effects are particularly relevant in complex, interrelated conditions such as heart failure with preserved ejection fraction, chronic kidney disease, atherosclerotic cardiovascular disease (ASCVD), metabolic dysfunction-associated steatotic liver disease (MASLD), and obesity. As such and despite barriers to use, GLP-1 RAs are increasingly recognized not only as agents for improving glycemic control and promoting weight loss, but as key components in the long-term management of metabolic and cardiometabolic disease (Fig. 1). This evolution in therapeutic use occurs against a backdrop of alarming trends in global obesity prevalence. In 2024, the World Health Organization reported that nearly 1 billion people worldwide are now living with obesity–a figure that underscores the urgent need for effective, scalable interventions to reduce associated morbidity and mortality [1, 2].

In this review, we examine the expanding role of GLP-1 RAs across the spectrum of metabolic disease. We explore their mechanisms of action, established and emerging clinical applications based on the growing body of literature supporting use (Table 1), and future potential–particularly in light of the need for integrated, patient-centered approaches to chronic disease prevention and management.

**Table 1.** **Key clinical trials of GLP-1 receptor agonists across therapeutic indications**.

Organ system	Randomized control trial	Drug name	Major findings	
Cardiovascular disease	FIGHT	Liraglutide	∙	No significant effect on the primary end point (mean rank 146 versus mean rank 156, *p* = 0.31)
			∙	Non-significant trend towards increased HF hospitalizations in treatment group (HR 1.30 [95% CI 0.89–1.88], *p* = 0.17)
	LIVE	Liraglutide	∙	No significant improvement in LVEF in the treatment group (mean change in LVEF –0.8% [95% CI –2.1–0.5], *p* = 0.24)
	EXSCEL	Exenatide	∙	No significant differences in major cardiovascular events between control and treatment groups (HR 0.91 [95% CI 0.83–1.0])
	STEP-HFpEF	Semaglutide	∙	Significant improvements noted in symptoms, exercise tolerance (6-minute walk distance difference 20.3 [95% CI 8.6–32.1], *p * < 0.001), inflammation (percent change in CRP estimated treatment ratio 0.61 [95% CI 0.51–0.72], *p * < 0.001), and quality of life (KCCQ-CSS estimated difference 7.8 points [95% CI 4.8–10.9], *p * < 0.001) in treatment group
			∙	Re-demonstrated benefit among patients with DM2 in STEP-HFpEF-DM2
	LEADER	Liraglutide	∙	Significantly lower rate of first occurrence of major cardiovascular event in the treatment group (primary composite outcome HR 0.87 [95% CI 0.66–0.93], *p* = 0.007)
	SUSTAIN-6	Semaglutide	∙	Significant reduction in major cardiovascular events in the treatment group (primary composite outcome HR 0.74 [95% CI 0.58–0.95], *p * < 0.001)
	SELECT	Semaglutide	∙	Significant reduction in major cardiovascular events in the treatment group (primary CV endpoint HR 0.80 [95% CI 0.72–0.90], *p * < 0.001)
			∙	Unlike SUSTAIN-6, SELECT participants did not have DM2
Renal outcomes	FLOW	Semaglutide	∙	Significant reduction in the treatment group (primary endpoint HR 0.76 [95% CI 0.66–0.88]; *p* = 0.0003)
Metabolic-dysfunction-associated steatotic liver disease	ESSENCE	Semaglutide	∙	Significantly more resolution of MASH in the treatment group (fibrosis resolution difference 28.7% [95% CI 21.1–36.2], *p * < 0.001
	SYNERGY-NASH	Tirzepatide	∙	Significantly more resolution of MASH in the treatment group for all doses (*p * < 0.001)
	LIVERAGE	Survodutide	∙	Met the primary end point of improvement in MASH with no worsening of fibrosis at 48 weeks (*p * < 0.001)
Obesity	SURMOUNT-1	Tirzepatide	∙	Significantly more weight loss in the treatment group for all doses (*p * < 0.001)
	STEP-1	Semaglutide	∙	Participants without diabetes achieved a mean weight reduction of 14.9% at 68 weeks compared to 2.4% with placebo (*p * < 0.001)
			∙	Redemonstrated benefit among patients with DM2 in STEP-2

HF, heart failure; HR, hazard ratio; CI, confidence interval; LVEF, left ventricular ejection fraction; CRP, C-reactive protein; KCCQ-CSS, Kansas City Cardiomyopathy Questionnaire clinical summary score; DM2, type 2 diabetes mellitus; CV, cardiovascular; MASH, metabolic dysfunction-associated steatohepatitis.

## 2. Weight Management

Weight loss is a mechanism through which GLP-1 RAs, at least in part, exert beneficial effects across organ systems. By acting on hypothalamic appetite-regulating centers, these agents reduce food intake, increase satiety, and slow gastric emptying and gut motility. The consequent reduction in visceral adiposity ameliorates insulin resistance, reduces systemic inflammation, and alleviates mechanical and metabolic stress on the heart, kidneys, and liver [3]. While GLP-1 RAs have direct systemic impact conferring benefit to these end organs as described in subsequent sections of this review, reducing epicardial adipose tissue (an essential driver of systemic inflammation) may reduce pericardial restraint, enhance ventricular interdependence, and improve right ventricular filling pressures [4, 5]. These systemic effects provide the foundation for the observed improvements in heart failure with preserved ejection fraction (HFpEF), chronic kidney disease (CKD), and MASLD outcomes in patients treated with GLP-1 RAs, which will be reviewed.

Clinical trial evidence strongly supports the efficacy of GLP-1 RAs in promoting weight loss among individuals with and without type 2 diabetes. The most robust data comes from the STEP (Semaglutide Treatment Effect in People with Obesity) trial program, which evaluated once-weekly semaglutide at a 2.4 mg dose in adults with overweight or obesity. In STEP 1, participants without diabetes achieved a mean weight reduction of 14.9% at 68 weeks compared to 2.4% with placebo (*p* < 0.001), alongside improvements in cardiometabolic risk factors. STEP 2, which included patients with type 2 diabetes, showed slightly lower weight loss (9.6% vs 3.4% with placebo) but confirmed significant glycemic benefits. The results highlight semaglutide’s effectiveness across populations, with the magnitude of weight loss varying based on metabolic status [6, 7].

SURMOUNT-1 (Tirzepatide Once Weekly for the Treatment of Obesity) investigated tirzepatide, a dual glucose-dependent insulinotropic polypeptide (GIP) receptor and GLP-1 RA, and demonstrated even more pronounced weight loss. At the lowest dose (5 mg weekly), participants lost an average of 15% of their body weight over 72 weeks, while participants who received the highest dose (15 mg weekly) lost an average of 20.9% of their body weight over 72 weeks compared to 3.1% with placebo (*p* < 0.001 for all comparisons with placebo) [8]. These findings underscore the growing potential of incretin-based therapies as pharmacologic tools for obesity management. Both semaglutide (Wegovy) and tirzepatide (Zepbound) are now approved by the Food and Drug Administration (FDA) for chronic weight management, and ongoing studies are evaluating long-term cardiovascular outcomes and durability of weight loss.

Ongoing efforts to expand obesity treatment options have led to the development of emerging combination therapies targeting additional mediators in the development of obesity, including GIP, glucagon, GLP-2 receptors, and amylin. One such therapy is retatrutide, a novel triple-hormone receptor agonist targeting GIP, GLP-1, and glucagon receptors. In a phase 2 trial with 338 participants, retatrutide demonstrated substantial dose-dependent weight loss, with reductions of 7.2% at 1 mg and 17.5% at 12 mg weekly over 24 weeks, compared to 1.6% with placebo. Cardiometabolic improvements–including better blood pressure, glycemic control, and lipid profiles–were observed at both 24 and 48 weeks. Notably, 72% of participants with prediabetes reverted to normoglycemia by week 48, and many were able to reduce or discontinue antihypertensive medications. Quality of life scores also improved in several domains, though without a consistent dose-response trend. The most common adverse events were gastrointestinal, largely occurring during dose escalation and mitigated by lower starting doses; the overall safety profile was consistent with other incretin-based therapies. Phase 3 trials are ongoing to optimize dosing strategies and further evaluate long-term safety and efficacy [9]. Another novel medication for weight management is dapiglutide, a combined GLP-1 RA and GLP-2 RA. While not yet published, the phase I DREAM trial reported topline results that included a mean weight loss of up to 4.3% after 12 weeks with treatment at a low dose and reasonable tolerability, with study completion estimated in August of 2025 [10]. There are several other exciting combination therapies at various stages of clinical investigation, including cagrilintide/semaglutide (GLP-1 RA and amylin antagonist), surodutide (GLP-1 RA and glucagon receptor agonist), and pemvidutide (GLP-1 RA and glucagon receptor agonist). Most trials estimate completion by 2027, and we anticipate that these results will continue to change the landscape of medical weight management [11].

Multiple randomized controlled trials with various agents have unequivocally demonstrated the effectiveness of GLP-1 RAs in weight loss and obesity, suggestive of a strong class effect. Nevertheless, further questions remain regarding discontinuation of therapy as well as long-term effects of therapy, including on body weight.

## 3. Cardiovascular Disease

Clinical trials evaluating GLP-1 RAs in heart failure have produced differing outcomes depending on heart failure subtype and severity, underscoring the importance of phenotype-specific therapy. Two key trials—FIGHT (Functional Impact of GLP-1 for Heart Failure Treatment) and LIVE (Liraglutide’s Influence on Ventricular Function in Chronic Heart Failure)—tested liraglutide in individuals with symptomatic heart failure with reduced ejection fraction (HFrEF). In FIGHT, inclusion criteria required a recent HF hospitalization within the prior 14 days with a pre-admission oral diuretic dose of at least 40 mg of furosemide. Both studies failed to demonstrate improvement in functional status, natriuretic peptide levels, or left ventricular remodeling among patients with HFrEF. In fact, the FIGHT trial showed a non-significant trend toward increased heart failure hospitalizations in the liraglutide group (95% CI 0.89–1.88; *p* = 0.17) [12, 13]. These findings were supported by a meta-analysis combining data from EXSCEL (Exenatide Study of Cardiovascular Event Lowering) [14] and FIGHT [12], which revealed an increased risk of heart failure hospitalization in HFrEF patients using GLP-1 RAs [15]. These outcomes suggest caution with GLP-1 RAs in advanced HFrEF, potentially due to the potential for adverse arrhythmic effects and HF decompensation. Another secondary analysis of the FIGHT trial suggested a trend towards atrial and ventricular arrhythmias with the use of liraglutide (predominantly atrial fibrillation and ventricular tachycardia) [16], a finding that was similar to the reported adverse events in the LIVE trial [13].

GLP-1 RAs are known to modestly increase heart rate, an effect that is thought to be mediated in part by calcium channel activity in cardiac pacemaker cells [17]. This chronotropic effect appears to be a class-wide phenomenon and is observed across several trials, including those evaluating liraglutide, semaglutide, and exenatide [18]. Mechanistically, GLP-1 receptors are expressed in the sinoatrial (SA) node, which is the heart’s primary pacemaker. When activated by GLP-1 RAs, these receptors can increase cyclic adenosine monophosphate (AMP) levels, which enhances the activity of L-type calcium channels and hyperpolarization-activated cyclic nucleotide-gated (HCN) channels. This then contributes to diastolic depolarization and pacemaker activity. Increased calcium influx through these channels leads to accelerated SA node firing, thereby increasing heart rate [17, 19]. While the magnitude of heart rate increase in clinical trials is generally small (around 2–5 bpm), this effect has raised concerns about its potential impact in patients with HFrEF, where elevated heart rate is associated with worse outcomes [20].

In contrast, GLP-1 RAs have demonstrated notable benefits in HFpEF, specifically in patients with obesity-related cardiac dysfunction. The STEP-HFpEF and STEP-HFpEF-DM trials evaluated semaglutide 2.4 mg weekly in individuals with HFpEF, with and without diabetes. Both trials showed clinically meaningful improvements in symptoms, exercise tolerance, and quality of life as measured by the Kansas City Cardiomyopathy Questionnaire (KCCQ); it was also associated with significant weight loss and reductions in NT-proBNP and inflammation (C-reactive protein) [21, 22]. In an echocardiographic substudy of the STEP-HFpEF trial, semaglutide was associated with favorable changes in cardiac structure and function, suggesting a potential disease-modifying effect in patients with obesity-related HFpEF. Compared with placebo, semaglutide significantly attenuated adverse cardiac remodeling, including reduced progression of left atrial enlargement (estimated mean difference in left atrial volume: –6.13 mL; *p* = 0.0013) and improvements in right ventricular size, as evidenced by reductions in both end-diastolic area (–1.99 cm^2^; *p* = 0.016) and end-systolic area (–1.41 cm^2^; *p* = 0.0064) [23].

Expanding beyond heart failure, the landmark SELECT trial provided robust evidence that GLP-1 RAs can reduce cardiovascular risk even in individuals without diabetes. Enrolling over 17,000 overweight or obese patients with established atherosclerotic cardiovascular disease, SELECT showed that semaglutide 2.4 mg weekly led to a 20% relative risk reduction in major adverse cardiovascular events (MACE), including cardiovascular death, nonfatal myocardial infarction, and nonfatal stroke, compared to placebo [24]. These benefits emerged independent of glycemic effects, and the Kaplan-Meier curves began to deviate before significant weight loss would be expected to occur.

A pre-specified analysis of the SELECT trial evaluated the effects of semaglutide versus placebo in patients with (n = 4286; 24.3%) and without heart failure, further subclassified into HFpEF, HFrEF, or unclassified types. Although baseline characteristics were generally similar, heart failure classifications were investigator-defined without standardized phenotyping. Semaglutide was associated with a 28% reduction in major adverse cardiovascular events (hazard ratio (HR) 0.72), a 21% reduction in the composite heart failure outcome (HR 0.79), a 24% reduction in cardiovascular death (HR 0.76), and a 19% reduction in all-cause mortality (HR 0.81), compared to placebo. These benefits were consistent across heart failure subgroups with no significant interactions by phenotype, although it is notable that 90% of SELECT participants had New York Heart Association (NYHA) class I or II functional status. The safety profile of semaglutide was similar between patients with and without heart failure. However, given limited power and prior inconsistent findings in HFrEF populations, further dedicated studies in this subgroup are warranted [25].

Taken together, these trials suggest that while GLP-1 RAs should be used with caution in HFrEF, especially those with a higher risk clinical profile such as the FIGHT population, they may offer substantial clinical benefits in patients with HFpEF and those with obesity and cardiovascular disease and risk factors, even in the absence of diabetes.

SURMOUNT-MMO is an ongoing randomized double-blind trial that will provide more robust cardiovascular outcomes associated with therapy with once-weekly tirzepatide injections [26]. The accumulating evidence positions GLP-1 RAs as a valuable addition to the therapeutic armamentarium for cardiometabolic disease, with phenotype-specific considerations playing a critical role in optimizing outcomes.

While a growing body of supportive data has increased the uptake of GLP-1RA therapy among patients with ASCVD and HFpEF, patient selection remains challenging, as demonstrated by a possible signal towards harm among those patients with HFrEF. While their use has been endorsed by guideline organizations in select patient populations, proper patient selection is paramount among an already multimorbid patient population.

## 4. Renal Outcomes

Though there is no approved renal indication for GLP-1 RAs, these agents have demonstrated consistent renal benefits across multiple large cardiovascular outcome trials despite renal outcomes being secondary endpoints in most studies. These benefits include reductions in albuminuria progression, preservation of estimated glomerular filtration rate (eGFR), and potential delayed onset of end-stage kidney disease. The proposed mechanisms include reduced glomerular hyperfiltration via natriuresis, attenuation of systemic and renal inflammation, and favorable effects on blood pressure, body weight, and glycemic control. Importantly, these effects appear to be independent of the glucose-lowering action of GLP-1 RAs [4].

Among the pivotal trials, LEADER (Liraglutide and Cardiovascular Outcomes in Type 2 Diabetes) demonstrated a 22% relative risk reduction in a composite renal outcome–primarily driven by a reduction in new-onset macroalbuminuria–in patients with type 2 diabetes and high cardiovascular risk [27, 28]. SUSTAIN-6 (Semaglutide and Cardiovascular Outcomes in Patients with Type 2 Diabetes) reported a 36% reduction in new or worsening nephropathy, also driven largely by reductions in albuminuria [29]. In contrast, an exploratory analysis of the REWIND trial (Dulaglutide and Cardiovascular Outcomes in Type 2 Diabetes), which included a broader population with lower cardiovascular risk, showed a 15% reduction in composite kidney outcomes with consistent benefits across eGFR subgroups [29, 30]. Notably, AMPLITUDE-O (Cardiovascular and Renal Outcomes with Efpeglenatide in Type 2 Diabetes) evaluated efpeglenatide (a once-weekly exendin-based GLP-1 RA) and demonstrated a 32% risk reduction in a prespecified composite kidney endpoint that included new macroalbuminuria, sustained eGFR decline, or need for dialysis. Importantly, these findings held true even in a cohort with advanced kidney disease and on top of background sodium–glucose cotransporter 2 (SGLT2) inhibitor use [31]. These findings suggest that renal benefits may be a class effect, though possibly more pronounced with agents that achieve greater weight loss and glycemic improvements.

Despite the encouraging findings, the absence of dedicated renal outcome trials for GLP-1 RAs has limited their positioning as frontline kidney-protective therapies. However, the ongoing FLOW trial, a randomized controlled trial of semaglutide in patients with type 2 diabetes and chronic kidney disease (eGFR 25–75 mL/min/1.73 m^2^ and urine albumin-to-creatinine ratio (UACR) ≥200 mg/g), is the first to evaluate renal outcomes as a primary endpoint [32].

Despite the encouraging findings, the absence of dedicated renal outcome trials for GLP-1 RAs has limited their positioning as frontline kidney-protective therapies. However, the FLOW trial, a randomized controlled trial of semaglutide 1 mg weekly vs placebo in patients with type 2 diabetes and chronic kidney disease (eGFR 50–75 mL/min/1.73 m^2^ and UACR >300 mg/g or eGFR 25–50 mL/min/1.73 m^2^ and UACR >100), is the first to evaluate renal outcomes as a primary endpoint [32]. The risk of the primary outcome–major kidney disease events (composite of onset of kidney failure, at least a 50% reduction in the eGFR from baseline, or death from kidney-related or cardiovascular causes)–was 24% lower in the semaglutide group than in the placebo group (*p* = 0.0003). Of note, all participants in the FLOW trial were required to be on maximally tolerated doses of renin angiotensin system inhibitors and were permitted to be on SGLT2 inhibitors (~15% of patients enrolled). The growing body of evidence in patients with risk factors or established chronic kidney disease may support broader adoption of GLP-1 RAs to improve renal outcomes.

The future of GLP-1 RA remains promising with regard to outcomes. Though multiple secondary analyses of large randomized controlled trials have demonstrated the safety and efficacy of therapy with GLP-1 RA, supportive data for this therapy purely for renal outcomes remains limited. And as of now, their use in CKD among guideline committees largely remains limited to patients with diabetes.

## 5. Use in Liver Disease

With the increasing burden of MASLD and metabolic dysfunction-associated steatohepatitis (MASH), GLP-1 RAs are being explored for their hepatic benefits. In a phase 2 trial by Newsome *et al*. [33], semaglutide 0.4 mg weekly led to MASH resolution in 59% of patients compared to 17% with placebo (*p* < 0.001), although without a significant difference in fibrosis improvement (43% vs 33% placebo; *p* = 0.48). These results laid the groundwork for larger and longer-term studies such as the ESSENCE trial (Phase 3 Trial of Semaglutide in Metabolic Dysfunction-Associated Steatohepatitis), which evaluated semaglutide’s effects on liver histology and clinical outcomes. In this phase 3 trial of 1197 adults with biopsy-confirmed non-cirrhotic MASH (fibrosis stages F1–F3), semaglutide 2.4 mg weekly achieved MASH resolution in 63% of participants versus 34% in the placebo group (*p* < 0.001), and ≥1-stage fibrosis improvement in 37% versus 22% (*p* < 0.001), respectively, after 72 weeks [34]. Together, these data suggest that semaglutide not only improves liver histology but may also offer disease-modifying potential in MASH, particularly when combined with its broader cardiometabolic benefits. Given their favorable metabolic profile and weight-reducing effects, GLP-1 RAs are emerging as promising agents in the evolving MASH treatment landscape.

Beyond semaglutide, newer incretin-based agents have shown even more striking effects. Tirzepatide, a dual GIP/GLP-1 receptor agonist, demonstrated MASH resolution in 44–62% of participants in the phase 2 SYNERGY-NASH trial as compared to 10% resolution in the placebo group (*p* < 0.001 across all dose comparisons) [35]. Survodutide, a GLP-1/glucagon receptor co-agonist, was assessed in a phase 2 trial and was shown to achieve MASH improvement in 43–62% and fibrosis improvement in 34–36% of participants over 48 weeks [36]. Retatrutide, a triple agonist (GIP, GLP-1, and glucagon), was also evaluated in a phase 2 trial and showed up to 82% relative liver fat reduction and normalization of liver fat content (<5%) in 86% of participants. These findings underscore a rapidly advancing therapeutic landscape in which incretin-based therapies–alone or in combination–are poised to transform the management of MASLD and MASH.

The utility of GLP-1 RA therapy in metabolic dysfunction-associated steatohepatitis is bolstered by multiple trials demonstrating histologic reversal of liver disease and modification of disease course. Though these therapies are not formally approved among patients with MASLD, they remain a promising therapy in a very modest armamentarium.

## 6. Additional Uses of GLP-1 RAs

Obstructive sleep apnea (OSA) is a frequent comorbidity of obesity, cardiovascular disease, renal impairment, and metabolic syndrome. A longstanding cornerstone of treatment of OSA has been continuous positive airway pressure (CPAP), though adherence with CPAP is estimated to be between 60–70% [37]. Untreated OSA can lead to significant morbidity and mortality, and has been associated with many adverse outcomes, including systemic and pulmonary hypertension, atherosclerotic cardiovascular disease, stroke, atrial fibrillation, and heart failure, among others [38]. The SURMOUNT-OSA trial (Tirzepatide for the Treatment of Obstructive Sleep Apnea and Obesity) showed an estimated treatment difference in apnea hypopnea index (AHI) of –20 events per hour as compared to placebo in those patients who were unwilling and/or unable to use positive airway pressure (PAP) therapy and treated with maximally tolerated doses of tirzepatide (*p* < 0.001). This study showed an even greater difference in AHI for those patients who had been using PAP therapy for at least 3 consecutive months at baseline (–23.8 events per hour; *p* < 0.001) [39]. A recent meta-analysis found that in adults with moderate to severe OSA, GLP-1 RAs significantly reduced the AHI by a mean of –10 events per hour, with tirzepatide demonstrating even greater reductions in AHI and body weight compared to liraglutide. Both agents also demonstrated a reduction in systolic blood pressure by an average of 5 mmHg [40]. It was these findings that led to the FDA approval of tirzepatide for the primary treatment of moderate to severe OSA in adults with obesity [41]. Similarly, GLP-1 RA therapy has been investigated with regard to the management of hypertension [42]. Sub-study analysis of SURMOUNT-1 demonstrated that tirzepatide therapy was associated with a reduction in 24-hour ambulatory blood pressure, and investigations with other incretin-based therapies, such as orforglipron, are ongoing [43, 44].

Emerging research has begun to investigate the potential role of GLP-1 RAs in the treatment of neurocognitive disorders, including Alzheimer’s disease and related dementias. GLP-1 receptors are expressed in the brain, and preclinical studies suggest that GLP-1 RAs may exert neuroprotective effects through anti-inflammatory mechanisms, promotion of neuronal survival, enhancement of synaptic plasticity, and stimulation of neurogenesis. In animal models, GLP-1 receptor activation has been shown to reduce amyloid plaque burden–a key pathological feature of Alzheimer’s disease–and improve learning and memory performance [22]. While human data remain limited and largely exploratory, early-phase studies using agents such as liraglutide and semaglutide have shown preliminary signals of cognitive benefit, particularly among patients with type 2 diabetes [23]. To more definitively assess these effects, two large-scale phase 3 trials, EVOKE and EVOKE Plus, are currently evaluating oral semaglutide in patients with early Alzheimer’s disease, with results expected to inform whether incretin-based therapies can offer disease-modifying effects in neurodegenerative conditions [45].

Despite emerging indications for the use of GLP-1 RAs, data within this space remains preliminary, and current FDA-approved indications for therapy include type 2 diabetes, cardiovascular risk reduction, and OSA (i.e., tirzepatide).

## 7. Guideline Recommendations

**Fig. 2.**
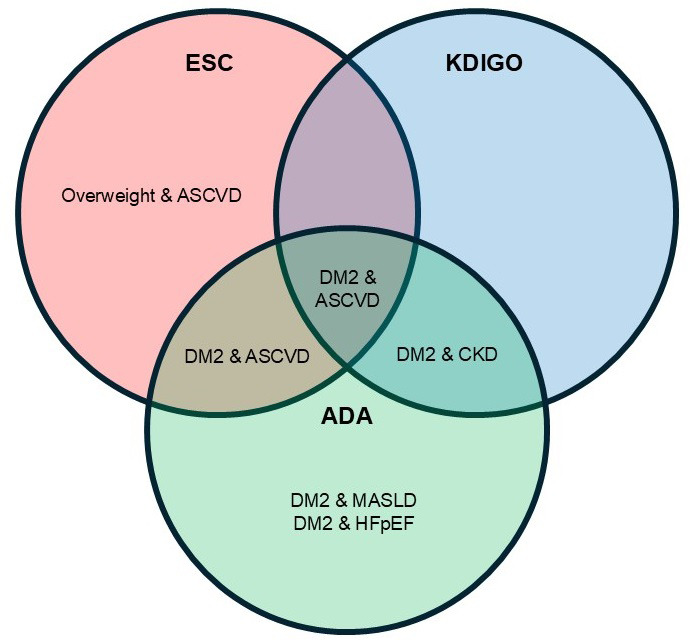
**Guideline-recommended indications for glucagon-like peptide-1 receptor agonists**. ASCVD, atherosclerotic cardiovascular disease; ESC, European Society of Cardiology; KDIGO, Kidney Disease Improving Global Outcomes; CKD, chronic kidney disease; ADA, American Diabetes Association; MASLD, metabolic dysfunction–associated steatotic liver disease; HFpEF, heart failure with preserved ejection fraction.

Although not all national guidelines have yet incorporated the latest evidence on the benefits of GLP-1 RAs, the most recent diabetes guidelines do support initiating GLP-1 RA therapy in appropriate, eligible patients to improve cardiovascular and renal outcomes (Table 2, Ref. [46, 47, 48]). Across all three guidelines, there is strong consensus that GLP-1 RAs should be prioritized for adults with type 2 diabetes who have obesity, cardiovascular disease, chronic kidney disease, or metabolic liver disease, due to their proven benefits in weight reduction, cardiovascular risk reduction, and glycemic control – independent of A1c and often preferred when SGLT2 inhibitors are contraindicated or insufficient. Though we anticipate these recommendations may evolve following the emergence of recent data, GLP-1 RAs remain central to comprehensive cardio-renal-metabolic management (Fig. 2).

**Table 2.** **Summary of contemporary guideline recommendations**.

National guideline	Class of recommendation/level of evidence	Recommendation
Obesity and cardiovascular disease: an ESC clinical consensus statement [46]	2A/B	Glucose-lowering medications with effects on weight loss (e.g., GLP-1RAs) should be considered in patients with DM2 with overweight or obese to reduce weight
	1/A	GLP-1RAs with proven cardiovascular benefit (liraglutide, subcutaneous semaglutide, dulaglutide, efpeglenatide) are recommended in patients with DM2 and atherosclerotic cardiovascular disease to reduce cardiovascular events, independent of baseline or target HbA1c and independent of concomitant glucose-lowering medication
	2A/B	The GLP-1 RA semaglutide should be considered in overweight (BMI >27 kg/m^2^) or obese chronic coronary syndrome patients without diabetes to reduce cardiovascular mortality, myocardial infarction, or stroke
KDIGO 2024 clinical practice guideline for the evaluation and management of chronic kidney disease [47]	1/B	In adults with DM2 and CKD who have not achieved individualized glycemic targets despite use of metformin and SGLT2 inhibitor treatment, or who are unable to use those medications, recommend a long-acting GLP-1 RA
American diabetes association standards of care in diabetes–2025 [48]	Cardio-renal Recommendations	
	A	In adults with DM2 and established or high risk of atherosclerotic cardiovascular disease, the treatment plan should include medications with demonstrated benefits to reduce cardiovascular events (e.g., GLP-1 and/or SGLT2 inhibitor) for glycemic management and comprehensive cardiovascular risk reduction (irrespective of A1c)
	A	In adults with DM2 and symptomatic HFpEF and obesity, a GLP-1 RA with demonstrated benefits for both glycemic management and reduction of HF-related symptoms should be used (irrespective of A1c)
	A	In adults with DM2 who have CKD (with confirmed eGFR 20–60 mL/min/1.73 m^2^ and/or albuminuria), an SGLT2 inhibitor or GLP-1 RA with demonstrated benefit in this population should be used for both glycemic management (irrespective of A1c) and for slowing progression of CKD and reduction in cardiovascular events. The glycemic benefits of SGLT2 inhibitors are reduced at eGFR <45 mL/min/1.73 m^2^
	B	In adults with DM2 and advanced CKD (eGFR <30 mL/min/1.73 m^2^), a GLP-1 RA is preferred for glycemic management due to a lower risk of hypoglycemia and for cardiovascular event reduction
	MASLD and MASH Recommendations	
	B	In adults with DM2, MASLD, and overweight or obesity, consider using a GLP-1 RA or a dual glucose-dependent insulinotropic polypeptide (GIP) and GLP-1 RA with potential benefits in MASH for glycemic management and as an adjunctive to healthy interventions for weight loss
	B	In adults with type 2 diabetes and biopsy-proven MASH or those at high risk for liver fibrosis (based on noninvasive tests), pioglitazone, a GLP-1 RA, or a dual GIP and GLP-1 RA is preferred for glycemic management due to potential beneficial effects on MASH

ESC, European Society of Cardiology; DM2, type 2 diabetes mellitus; HbA1c, hemoglobin A1c; BMI, body mass index; KDIGO, Kidney Disease Improving Global Outcomes; CKD, chronic kidney disease; SGLT2, sodium–glucose cotransport 2; HFpEF, heart failure with preserved ejection fraction; HF, heart failure; eGFR, estimated glomerular filtration rate; MASLD, metabolic dysfunction–associated steatotic liver disease; MASH, metabolic dysfunction–associated steatohepatitis.

## 8. Translating Evidence to Implementation

Despite their broad potential, GLP-1 RAs often remain prohibitively expensive, and access is limited by insurer-imposed restrictions, especially for indications beyond type 2 diabetes. A 2022 policy analysis by the Institute for Clinical and Economic Review (ICER) concluded that current pricing for GLP-1 RAs exceeds traditional cost-effectiveness thresholds for obesity treatment, though prices were more favorable when cardiovascular and renal benefits were included [49]. As of April 2025, Medicare Part D provides coverage for GLP-1 RAs, including semaglutide (Ozempic) and liraglutide (Victoza) for FDA-approved indications such as type 2 diabetes mellitus and cardiovascular risk reduction in select high-risk populations. However, these agents are not currently reimbursed by Medicare when prescribed solely for the treatment of obesity or weight management despite growing evidence supporting their efficacy in reducing obesity-related comorbidities. This limitation is rooted in existing statutory provisions that prohibit Medicare coverage for drugs indicated exclusively for weight loss, regardless of their broader health benefits [50].

In late 2024, the Biden administration proposed expanding Medicare and Medicaid coverage to include GLP-1 RAs for obesity treatment, citing their potential to improve population health and reduce long-term expenditures due to obesity-associated diseases. However, in April 2025, the Centers for Medicare & Medicaid Services (CMS) declined to move forward with the proposed change, maintaining the current exclusion of anti-obesity pharmacotherapy. This decision holds considerable implications for public health and healthcare policy, with recent estimates suggesting that nearly 40% of Medicare beneficiaries meet eligibility criteria for GLP-1 RA therapy based on body mass index (BMI) and associated risk factors. Modeling studies project that broader access could yield substantial cost savings over time by reducing cardiovascular events, hospitalizations, and other obesity-related complications [51].

Adherence challenges and high discontinuation rates also complicate implementation. Real-world evidence from implementation studies suggests high dropout rates due to gastrointestinal adverse events, medication cost, and difficulty with long-term adherence. A recent study by Gleason *et al*. [52] analyzed real-world data on GLP-1 RAs among commercially insured adults without diabetes. The average adherence determined by proportion of days covered during the 1-year assessment was 51%, with only 27% of patients having an adherence rate of ≥80%. While they did not study this, anecdotally, the authors noted adverse effects and cost most commonly contribute to discontinuation rates [52]. Moreover, patient selection must be refined to ensure therapy is reserved for those likely to derive meaningful clinical and economic benefit. From a value-based care perspective, targeting GLP-1 RAs to patients with high cardiometabolic risk–including those with obesity, diabetes, and cardiovascular or renal comorbidities–may offer long-term cost-savings that are not captured in one-year cycles under which most payers operate. The delayed cost-saving impact of GLP-1 RAs thereby limits the incentive to facilitate the prescription of a high-cost medicine in hopes of future risk reduction.

Implementation science studies also highlight barriers to integration into clinical practice, including clinical inertia, reluctance to polypharmacy, perceived titration complexity, and insurance coverage limitations (Table 3). These barriers undermine initiatives that prioritize long-term outcomes and preventive strategies. Recent efforts to optimize the use of GLP-1 RAs in clinical practice have included the development of pharmacist-led titration services aimed at improving medication management and mitigating therapeutic inertia [53]. One study evaluated the implementation of such a service, where pharmacists were responsible for guiding dose titration and monitoring for adverse effects [54]. This model facilitated improved medication access, more frequent patient follow-up, and comprehensive device and lifestyle management education, ultimately supporting improved adherence and outcomes among patients initiating GLP-1 RA therapy for weight loss.

**Table 3.** **GLP-1 receptor agonists: summary of benefits and barriers**.

Benefits	Barriers/Unknowns
Robust weight loss	High drug cost; insurance restrictions
Reduction in major adverse cardiovascular events	Neutral or adverse findings in HFrEF; unclear long-term safety in advanced HF
Improvements in HFpEF symptoms, function, and cardiac remodeling	Mechanisms of benefit vs harm across HF phenotypes remain uncertain
Kidney protection	Limited head-to-head data vs SGLT2 inhibitors; additive or synergistic benefit needs confirmation
Histologic resolution of MASH and fibrosis improvement	Durability of benefit after discontinuation is unknown
FDA approval of tirzepatide for obstructive sleep apnea	Adherence challenges: GI intolerance, discontinuation >40% in real-world studies
Once-weekly dosing improves convenience	Medicare exclusion for obesity treatment; cost-effectiveness
Population-level potential to reduce cardiometabolic burden; aligns with value-based care goals	

FDA, Food and Drug Administration; HFrEF, heart failure with reduced ejection fraction; GI, gastrointestinal.

In addition to care team restructuring, quality improvement initiatives have also demonstrated success in increasing the uptake of GLP-1 RAs and SGLT2 inhibitors among high-risk individuals with type 2 diabetes and comorbid conditions such as atherosclerotic cardiovascular disease, chronic kidney disease, or heart failure. One such intervention incorporated provider education, clinical decision support, and audit-feedback mechanisms to influence prescribing behavior. The initiative led to significant improvements in the prescription of evidence-based therapies for cardiometabolic disease prevention and management, illustrating the value of system-level changes in driving guideline-concordant care [55].

## 9. Oral GLP-1 Receptor Agonists

Orforglipiron, an oral GLP-1 agonist, has been shown to lower hemoglobin (Hgb) A1C by 1.3–1.6% from a baseline of 8%; more than 65% of participants who tolerated the highest dose achieved an A1C less than 6.5%. ACHIEVE-1, a phase 3 clinical trial, also reported 7.9% weight loss among participants at the highest dose of orfoglipiron. This medication is taken as a once daily oral medication and requires no dietary or fluid restrictions [56]. This is in contrast to oral semaglutide, which must be taken on an empty stomach and with minimal fluid intake. Oral semaglutide has been shown to lower Hgb A1C without the added benefit of weight loss and has shown non-inferiority for cardiovascular outcomes as compared to placebo [57]. Currently, orfoglipiron has not been approved by the FDA for diabetes or weight management, while oral semaglutide (Rybelsus) is FDA approved for diabetes management [58]. Despite the fact that oral therapies require daily administration and data is limited across the spectrum of cardiorenal metabolic disease, the expansion to oral formulations may offer a more convenient, non-injectable option that may improve adherence and broaden access for patients who are hesitant or unable to use injectable medications.

## 10. Pharmacokinetic and Clinical Considerations

GLP-1 RAs can influence the pharmacokinetics of other medications primarily through their effects on gastrointestinal motility. By delaying gastric emptying, GLP-1 RAs may alter the maximum concentration and time to maximum concentration of orally administered drugs, potentially impacting their efficacy or safety profile. This is especially relevant for medications with a narrow therapeutic index, such as warfarin, digoxin, carbamazepine, tacrolimus, and levothyroxine. For instance, studies have shown that liraglutide and exenatide can slow the rate–but not necessarily the extent–of drug absorption, leading to delayed peak plasma concentrations of co-administered agents [59, 60]. Clinical monitoring, including testing associated with efficacy and safety of co-administered agent and close monitoring of drug levels when available, and possible dose adjustments may be required when initiating or escalating GLP-1 RA therapy in patients on such medications, and further clinical studies are needed to define the impact.

In addition to delayed gastric emptying, nausea and vomiting–common side effects of GLP-1 RAs–can further compromise drug absorption and adherence. Patients should be counseled on dietary modifications to enhance the tolerability of GLP-1 RAs, including shifting to smaller, more frequent meals and eating slowly to allow natural satiety cues to take effect. Emphasis should be placed on adequate hydration and incorporating high-protein foods and fresh produce. Because high-fat meals can further delay gastric emptying and worsen gastrointestinal side effects, choosing lower-fat options may help mitigate these symptoms and improve overall treatment adherence. Moreover, the interaction may be more pronounced with short-acting GLP-1 agonists (e.g., exenatide twice daily), which have greater effects on gastric motility compared to long-acting agents like dulaglutide or semaglutide [61]. As the use of GLP-1 agonists expands into populations with polypharmacy, such as those with cardiovascular, renal, or transplant comorbidities, clinicians should be vigilant about potential pharmacokinetic interactions and prioritize medication reconciliation and individualized risk assessments.

Additional concerns with GLP-1 RAs include the potential for weight regain after discontinuation and their effects on muscle mass and function. A meta-analysis found significant weight regain post-treatment with GIP and GLP-1 RAs, likely due to the reversal of mechanisms such as appetite suppression, delayed gastric emptying, and increased energy expenditure [62]. In the SURMOUNT-4 randomized clinical trial, adults with obesity or overweight (without diabetes) achieved a mean weight loss of 20.9% after 36 weeks of open-label treatment with the maximum tolerated dose of tirzepatide. However, participants who were switched to placebo at week 36 experienced a 14% weight regain by week 52. In contrast, those who continued tirzepatide therapy experienced an additional 5.5% weight loss over the same period [63]. These findings highlight the importance of implementing and sustaining lifestyle modifications during initiation of these agents. Additionally, clinical trials show varied impacts on lean mass, with losses ranging from 25% to 45% of total weight lost, though lean mass reductions do not always indicate loss of muscle [6, 8]. Some evidence suggests that muscle quality may improve, but careful consideration is still needed for older or frail individuals. Future research should focus on evaluating muscle function and developing strategies to preserve muscle health during weight loss.

Though the link between diabetic retinopathy (DR) and GLP-1 RA is not fully understood, clinical trials have suggested that there may be an association between DR and GLP-1 RA. Findings from LEADER and SUSTAIN-6 demonstrated a higher incidence of DR-associated events among their treatment groups [27, 29]. The relationship between DR and GLP-1 RA remains unclear, and there is no formal clinical guidance on recommended retinal screening among patients receiving GLP-1 RA therapy. Based on a meta-analysis, GLP-1 RA use was associated with increased risk for early-stage DR and was protective when compared to insulin against late-stage DR [64].

With the increasing use of GLP-1 RAs, clinicians should also be aware of the periprocedural management of these medications. Due to the aforementioned effects on delayed gastric emptying, there is a hypothesized increased risk of aspiration during endotracheal intubation or deep sedation, though the data for this is limited to case reports and small retrospective studies [65]. With this in mind, the American Society of Anesthesiologists Task Force initially recommended that GLP-1 RAs should be held either one day (for daily medications) or one week (for weekly medications) prior to a planned procedural intervention. However, these recommendations were more recently revised to include a focus on shared decision-making and patient risk stratification to identify low-risk patients who may safely continue GLP-1 RAs pre-procedurally [66].

## 11. Conclusion 

GLP-1 receptor agonists represent a paradigm shift in chronic disease management. Their growing indications now span cardiovascular health, obesity, renal disease, and liver disease. There remain many questions surrounding this therapy, especially in combination with other glucose-lowering agents such as SGLT2 inhibitors. GLP-1 receptor agonists remain nascent, and the long-term effects of this therapy are not fully understood within the context of efficacy that is contingent on adherence. Future research should clarify the benefits and safety of this therapy among patients without diabetes mellitus but with high cardiovascular, hepatic, and renal risk factors. Despite the evident cardiometabolic effects of GLP-1 RA, considerable ambiguity regarding their role in select patient populations remains, namely among those with heart failure with reduced ejection fraction and diabetic retinopathy. Lastly, as the popularity of this drug soars, policy will need to target cost and affordability as these factors are major barriers to accessibility.

As evidence expands, addressing issues of affordability, access, and integration into value-based care models will be critical to unlocking the full population-level benefits of these therapies.

## Abbreviations

GLP-1 RA, glucagon-like peptide-1 receptor antagonist; MASLD, metabolic dysfunction-associated steatotic liver disease; ASCVD, atherosclerotic cardiovascular disease; HFpEF, heart failure with preserved ejection fraction; CKD, chronic kidney disease; GI, gastrointestinal; STEP, Semaglutide Treatment Effect in People with Obesity; SURMOUNT, Tirzepatide Once Weekly for the Treatment of Obesity; GIP, glucose-dependent insulinotropic polypeptide; GLP-2 RA, glucagon-like peptide-2 receptor antagonist; FIGHT, Functional Impact of GLP-1 for Heart Failure Treatment; LIVE, Liraglutide’s Influence on Ventricular Function in Chronic Heart Failure; CI, confidence interval; EXCSEL, Exenatide Study of Cardiovascular Event Lowering; HFrEF, heart failure with reduced ejection fraction; SA, sinoatrial; AMP, adenosine monophosphate; HCN, hyperpolarization-activated cyclic nucleotide-gated; KCCQ, Kansas City Cardiomyopathy Questionnaire; KCCQ-CSS, Kansas City Cardiomyopathy Questionnaire clinical summary score; cm, centimeter; MACE, major adverse cardiovascular event; HR, hazard ratio; NYHA, New York Heart Failure Association; eGFR, estimated glomerular filtration rate; LEADER, Liraglutide and Cardiovascular Outcomes in Type 2 Diabetes; SUSTAIN, Semaglutide and Cardiovascular Outcomes in Patients with Type 2 Diabetes; REWIND, Dulaglutide and Cardiovascular Outcomes in Type 2 Diabetes; AMPLITUDE-O, Cardiovascular and Renal Outcomes with Efpeglenatide in Type 2 Diabetes; SGLT2, sodium–glucose cotransporter 2; mL, milliliter; min, minute; m, meter; UACR, urine albumin-to-creatinine ratio; ESSENCE, Phase 3 Trial of Semaglutide in Metabolic Dysfunction-Associated Steatohepatitis; OSA, obstructive sleep apnea; CPAP, continuous positive airway pressure; SURMOUNT-OSA, Tirzepatide for the Treatment of Obstructive Sleep Apnea and Obesity; AHI, apnea hypopnea index; PAP, positive airway pressure; FDA, Food and Drug Administration; DM2, type 2 diabetes mellitus; CMS, Centers for Medicare & Medicaid Services; BMI, body mass index; hgb, hemoglobin; HF, heart failure; HR, hazard ratio; CI, confidence interval; LVEF, left ventricular ejection fraction; CRP, C-reactive protein; CV, cardiovascular; MASH, metabolic dysfunction-associated steatohepatitis; ESC, European Society of Cardiology; HbA1c, hemoglobin A1c; BMI, body mass index; KDIGO, Kidney Disease Improving Global Outcomes; CKD, chronic kidney disease; MASLD, metabolic dysfunction–associated steatotic liver disease.
